# Evaluation of Bortezomib-BeEAM (2BeEAM) as Chemotherapy Regimen Prior to ASCT in Patients with Mantle Cell Lymphoma

**DOI:** 10.3390/cancers15072091

**Published:** 2023-03-31

**Authors:** Fabrizio Huwyler, Rebekka Kunz, Ulrike Bacher, Michèle Hoffmann, Urban Novak, Michael Daskalakis, Yara Banz, Thomas Pabst

**Affiliations:** 1 Department of Medical Oncology, Inselspital, Bern University Hospital, 3010 Bern, Switzerland; 2 Department of Hematology and Central Hematology Laboratory, Inselspital, Bern University Hospital, University of Bern, 3010 Bern, Switzerland; 3 Institute of Pathology, Inselspital, University of Bern, 3008 Bern, Switzerland

**Keywords:** high-dose chemotherapy, bortezomib, BeEAM, mantle cell lymphoma, feasibility and toxicity

## Abstract

**Simple Summary:**

In patients with mantle cell lymphoma (MCL), an effective and safe high-dose chemotherapy regimen as consolidation of first-line treatment prior to ASCT is a matter of ongoing exploration due to unsatisfying outcomes with a relevant rate of relapse in the following course of disease. In our study, we evaluated the use of bortezomib together with standard HDCT of BeEAM (2BeEAM) in 11 patients with an MCL in a single institution study, assessing feasibility, toxicities, and survival rates. More than half of the patients presented with peripheral sensory and/or motor neuropathy. Progression-free survival and overall survival were 64% after a median follow-up time of 22 months. Moreover, 55% of the patients were in complete remission. These findings suggest the addition of bortezomib is associated with substantial toxicities without having a superior benefit in MCL therapy. Thus, we could not identify an obvious clinical benefit compared to standard treatment.

**Abstract:**

(1) Background: First-line therapy in fit MCL patients may comprise high-dose chemotherapy (HDCT) with autologous transplantation to consolidate remission before maintenance treatment. However, optimization of HDCT is an unmet clinical need given the substantial relapse rate of first-line treatment, while the use of bortezomib is a promising candidate to be added to standard HDCT. (2) Methods: We analyzed 11 consecutive patients with MCL who received bortezomib added to standard BeEAM (2BeEAM) HDCT at a single academic institution. We assessed safety, feasibility, toxicities, and survival rates. (3) Results: All patients had stage III or IV disease. We found that six patients (55%) developed new or worsening of preexisting peripheral neuropathy following administration of 2BeEAM HDCT. One patient relapsed within the first six months after HDCT, whereas three patients never reached complete remission. After a median follow-up of 22 months, the PFS was 64% and the OS 64% at the last follow-up assessment. At this time, 55% of patients were in CR. (4) Conclusions: The use of bortezomib added to standard BeEAM HDCT is associated with relevant toxicities, particularly with regards to additional neuropathy. Moreover, the anti-lymphoma efficacy of 2BeEAM HDCT appears to be modest; therefore, other therapeutic options should be evaluated for consolidation in this patient group.

## 1. Introduction

Mantle cell lymphoma is a rare subtype of lymphoma and accounts for 5–7% of malignant lymphomas, with a total incidence of 1-2/100′000. The hallmark of this lymphoma is the translocation t(11;14)(q13;q32), which results in an overexpression of cyclin D1 [1,2]. Mantle cell lymphoma is typically characterized by an aggressive clinical course with a median overall survival of approximately 4–5 years. The majority of patients diagnosed with mantle cell lymphoma are at an advanced stage of disease (stages III–IV) [3].

For young patients with mantle cell lymphoma, immune-chemotherapy such as three cycles with R-CHOP alternating with three cycles of a regimen containing cytarabine like R-ESAP or R-DHAP followed by HDCT and ASCT and followed by rituximab maintenance is considered a standard for first-line treatment [4]. In relapsed or refractory MCL, mTOR- and bruton-tyrosine-kinase (BTK) inhibitors are used such as ibrutinib. However, these current strategies are not curative in most patients with MCL, and PFS rates in relapsed or refractory MCL patients decrease with each treatment line [5,6].

The BEAM regimen (carmustine, etoposide, cytarabine, and melphalan) is a widely used conditioning regimen prior to ASCT. The adapted BeEAM regimen substituting carmustine with bendamustine showed a promising PFS [7,8] while being associated with relevant toxicities such as acute renal failure [8]. These facts urge further development of more effective and less toxic HDCT regimens [9].

Bortezomib, a proteasome inhibitor, showed activity in relapsed/refractory MCL [10,11]. In two studies by Robak et al., bortezomib replaced vincristine in the R-CHOP regimen as frontline therapy for MCL in patients who were not eligible for stem cell therapy. The bortezomib regimen was found to be more effective than vincristine at the cost of higher toxicity and showed a significantly longer survival in the bortezomib group while presenting a manageable safety profile even in the final overall survival follow-up after a median time of 82 months [12,13]. Bortezomib could also show a benefit in PFS as maintenance or consolidation therapy in ASCT-eligible patients with MCL [14]. Given these very promising results confirming the effectiveness of bortezomib, it was obvious to study its properties in an adapted therapeutic context. In this study, we aimed to investigate whether an addition of bortezomib may provide a benefit to the established BeEAM regimen in curative therapy prior to ASCT.

## 2. Materials and Methods

We conducted a retrospective case-series study at the University Hospital Inselspital, Bern, Switzerland. The cohort included all patients with mantle cell lymphoma who were treated at our university hospital with bortezomib, bendamustine, cytarabine, etoposide, and melphalan (2BeEAM) regimen as HDCT followed by ASCT. Inclusion criteria were that patients needed to be diagnosed with MCL and be at least in partial remission to be eligible for ASCT. A minimal number of 2.0 × 10^6^ CD34 + cells/kg b.w. was required. Patients had to be at a minimum age of 18 years and have a performance status of ECOG 0-1 to be included in the study. The diagnosis of MCL was based upon histomorphology and immunochemistry, and further by fluorescence in-situ hybridization (FISH), in the case of bone marrow infiltration, as well as multiparameter flow cytometry. These patients were the first ones to receive 2BeEAM. Treatments took place from January 2020 to June 2021.

The components of the 2BeEAM HDCT were based on the standard dosage of the BeEAM regimen. The doses were almost identical to those in the large study published by Prediletto et al. [15]. The only variation was a slightly higher dose of etoposide in our study. Prediletto et al. dosed etoposide 200 mg/m^2^ per day [15]. The dosage of bortezomib (1.3 mg/m^2^) was in accordance with the standard dosage used in daily practice. The applied dose was administered in previous studies showing activity and promising results in MCL patients [10,12]. Our 2BeEAM chemotherapy consisted of intravenous (i.v.) bendamustine 200 mg/m^2^ body surface (b.s.) on days -7 and -6. Subcutaneous (s.c.) injection of bortezomib 1.3 mg/m^2^ b.s. was carried out on days -7, -4, and -1. Etoposide was infused i.v. in a dosage of 150 mg/m^2^ b.s. on days -5, -4, -3, and 2 twice daily. Cytarabine 200 mg/m^2^ b.s. was administered on days -5, -4, -3, and -2 twice daily. The regimen ended with melphalan 140 mg/m^2^ b.s. on day 1. Eventually, ASCT was performed on day 0. The patients received G-CSF (filgrastim) in a weight-adjusted dosage of 5 μg/kg body weight. It was administered from day +6 after ASCT for at least eleven days and until neutrophils exceeded 0.5 g/L for three consecutive days. The patients received an antifungal prophylaxis with sulfamethoxazole 800 mg/trimethoprim 160 mg, three times per week, and fluconazole 400 mg weekly. Antiviral prophylaxis consisted of valaciclovir 500 mg. No antibacterial prophylaxis was given. We assessed age, stage, B-symptoms, the Ki-67 index, the MCL international prognostic index (MIPI), remission status at the time of ASCT, remission status at follow-up, and subsequent hematological and non-hematological toxicities. We used the CTCAE version 5.0 score to grade hematological toxicities.

Progression-free (PFS) and overall survival (OS), by the Kaplan–Meier method, were defined as the time from HDCT to disease progression or death. PFS and OS were censored at the last follow-up on 1st of March 2023, which was also used as the data cutoff.

For the figures, we used GraphPad Prism version 8. Categorical variables were summarized as frequencies and percentages, and the continuous variables were summarized as medians and ranges.

## 3. Results

### 3.1. Clinical Characteristics of the Patients

The clinical characteristics of the 11 patients are summarized in Table 1. The median age of the patients at the time of diagnosis was 66 years. All patients were diagnosed with mantle cell lymphoma—8 patients with the classical type and in 2 patients with the blastoid variant. At diagnosis of the MCL, the patients all showed an advanced stage—stage III in 3 patients (27%, 3/11) or stage IV in 8 patients (73%, 8/11). The average Ki-67 proliferation index was 36% (16.6) and therefore above the 30% cutoff for an unfavorable clinical course.

### 3.2. Clinical Status at the Time of HDCT/ASCT

We present the clinical status at the time of HDCT/ASCT in Table 2. At the time of HDCT/ASCT, 6 patients had a partial remission (PR, 55%, 6/11) and 5 were in complete remission (CR, 45%, 5/11). The majority of patients were in the intermediate or high MIPI risk category. Only one patient was in the MIPI low-risk category. Six patients (55%, 6/11) suffered from clinically important comorbidities, assessed with the hematopoietic cell transplantation specific comorbidity index (HCT-CI), while all patients had ECOG status 0.

### 3.3. Toxicities and Adverse Events Following HDCT/ASCT

The median hospitalization time after HDCT and ASCT was 27 days. Predominantly hematologic toxicities were seen with all of the patients suffering from anemia with a corresponding CTCAE grade 3 in median, febrile neutropenia and thrombocytopenia both with a median of CTCAE grade 4, whereby all of the patients had preexisting thrombocytopenia and anemia.

Peripheral neuropathies that were either new or intensified were documented in six patients. Five patients suffered from peripheral sensory neuropathy with a median of CTCAE grade 2, which was in one case combined with a motoric neuropathy (CTCAE 2). The sixth patient suffered from isolated motoric neuropathy CTCAE 3. Neurologic deficiencies had completely resolved in 5/6 patients after at least six months, whereas in 1/6 patients the neuropathy had not completely resolved.

Hypotension, rash, and dyspnea were seen in more than half of the patients. Other unspecific but typical adverse events such as nausea, fatigue, constipation, diarrhea, and weight loss were seen in the majority of the patients, as listed in Table 3 below.

### 3.4. Clinical Course after HDCT/ASCT

At a 100-day follow-up (Table 4), seven patients were in complete remission, three were in partial remission, and one patient had died in the meantime due to a cardiac event. At the last follow-up, which was at a median time of 22 months after HDCT/ASCT, six patients were in complete remission, one patient was in partial remission, and three more patients died. One of them died after a relapse, whereas he had initially achieved complete remission. The other two never achieved complete remission after HDCT/ASCT. Nine patients received additional anti-lymphoma treatment in the later clinical course after HDCT/ASCT. Seven patients received rituximab, one patient received ibrutinib, and one patient received both rituximab and ibrutinib. In total, rituximab was administered to all eight patients as maintenance therapy after HDCT/ASCT. The administration of ibrutinib in two patients was initiated after a relapse in one patient and in a refractory MCL in another patient.

**Figure 1:**
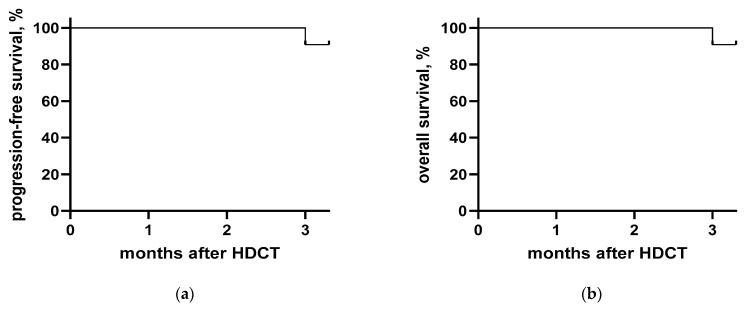

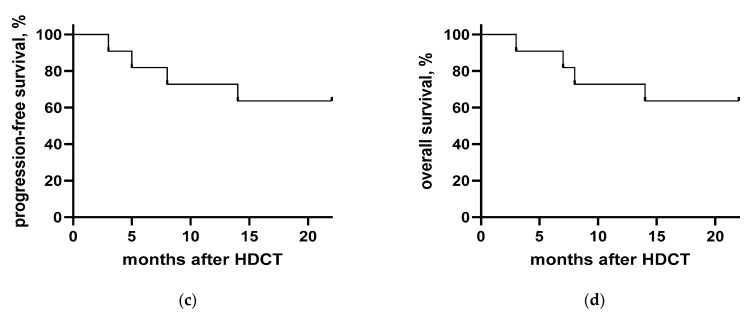
(**a**) Progression-free and (**b**) overall survival at a 100-day follow-up after HDCT/ASCT in patients who received Bortezomib-BeEAM. (**c**) Progression-free and (**d**) overall survival at the last assessed follow-up at a median of 22 months after HDCT/ASCT in patients who received bortezomib.

Figure 1 depicts the survival curves of the patients after they received HDCT/ASCT. The progression-free survival and overall survival at the 100-day follow-up (Figure 1a,b) show only one patient with progression, respectively, death. However, considering the results of the last follow-up, which took place at a median of 22 months after the HDCT/ASCT (Figure 1c,d), progression of the disease was documented in three patients after five, eight, and fourteen months. This sums up to a progression-free survival rate of 64% at the last assessed follow-up. Within the interval between the 100-day follow-up and the last follow-up, three more mortalities were observed. This sums up to an overall survival rate of 64% at the last follow-up.

## 4. Discussion

In eligible patients, HDCT with the BeEAM regimen followed by ASCT is currently the established first-line treatment and has a positive effect on outcomes in MCL patients. However, limited improvements in overall survival and relevant toxicities remain [4,7,8]. Therefore, we aim to provide an adapted HDCT regimen by adding bortezomib to obtain better outcomes. This retrospective study reports on the feasibility and toxicity of the adapted BeEAM regimen with added bortezomib as a conditioning regimen before ASCT in patients with MCL.

Since event-free and overall survival were found to be significantly improved by the concept of HDCT (carmustine, etoposide, cytarabine, cyclophosphamide (BEAC)) and ASCT compared to conventional chemotherapy in various entities of non-Hodgkin lymphoma, HDCT/ASCT became the preferred therapy option [16]. Nevertheless, relapse post-HDCT/ASCT remains a problem, which holds especially true for MCL patients, as relapses post-HDCT/ASCT are almost universal and the MCL is even considered incurable [17]. Whether the use of CD34+-selected autografts provides additional survival benefits in MCL patients remains unclear and needs prospective evaluation [18]. Little data is available to guide the selection through the various HDCT regimens prior to ASCT. A large retrospective study by Chen et al. from the Massachusetts General Hospital compared BEAM, CBV (cyclophosphamide, BCNU, and etoposide), BuCy, and TBI (total body irradiation)-containing regimens in various non-Hodgkin and Hodgkin lymphomas. CBV was divided into a “high” and “low” subgroup according to the dose of BCNU. This study could show the benefits of CBVlow in MCL patients [19]. Further studies suggest alternatives to BEAM such as BuCyE (busulfan, cyclophosphamide, etoposide) or TEAM (thiotepa, etoposide, cytarabine, melphalan) regimens [20,21], albeit with limited benefit.

Visani et al. conducted a phase I/II study with BeEAM HDCT/ASCT in resistant or relapsed non-Hodgkin and Hodgkin lymphoma patients. They reported a high CR rate of 81% after a median follow-up of 18 months. In an update, they could show a favorable 3-year PFS of 72% [22,23]. The BeEAM regimen aims to replace BCNU with bendamustine, thereby hopefully eliminating the pulmonary toxicities associated with BCNU. To avoid BCNU-related toxicities, we introduced the BeEAM regimen in our center in September 2013 as a standard conditioning regimen for lymphoma patients. In our previous study published by Gilli et al., we investigated the safety and effectiveness of the BeEAM regimen in 39 patients with different lymphomas [24]. With a CR of 74% and PFS after 2 years of 69%, the outcomes were similar to those reported by Visani et al. and supported their report of favorable outcomes compared to the corresponding survival rates of other HDCT regimens. [22,23,24]. There was some renal toxicity associated with bendamustine, which even led to one patient requiring transient dialysis. These toxicities were completely reversible within one week but seemed not to occur in patients receiving BEAM [9]. This suggests that bendamustine probably is the origin of increased renal toxicity, whereas pulmonary toxicities seemed to be a rare event under bendamustine [24]. Hence, BeEAM seems to be a promising alternative to BEAM.

Bortezomib was studied as part of chemotherapy regimens in MCL patients at relapse or ineligible for HDCT/ASCT as an addition to CHOP and could also show a benefit in frontline therapy with R-CHOP substituting bortezomib for vincristine [12,25]. Nevertheless, there are only a few studies investigating bortezomib as part of HDCT. William et al. investigated the effects of bortezomib added to BEAM in HDCT (V-BEAM) in 26 patients with MCL. There was no significant benefit in PFS or OS after a follow-up of 1 and 5 years [26], but the number of patients was limited, and the study was not randomized. Therefore, further investigation of bortezomib as part of HDCT is justified, which was finally the motivation for our study evaluating bortezomib as an addition to BeEAM (2BeEAM) in the context of HDCT prior to ASCT in MCL patients.

In our patient cohort, all patients had MCL stage III or IV; 63.6% had B-symptoms; the median age was 66 years; and the male-to-female ratio was 2.7:1. Thus, although the size of the cohort was limited, all relevant characteristics were matched with data from the literature [27]. The demographic characteristics of our here presented cohort receiving bortezomib-BeEAM were well matched with the characteristics of our previous study cohort receiving BeEAM (without bortezomib) at our center, as published by Gilli et al. [24]. The patients receiving bortezomib-BeEAM in our present study spent a median of 27 days in hospital, which was in perfect correlation with the results of Gilli et al. Whereas neutrophil engraftment was identical, with a median of 11 days in the cohort of Gilli et al., the median time to thrombocyte engraftment was considerably longer in our present study, with 22 days compared to 15 days as reported by Gilli et al. All patients in our bortezomib-BeEAM cohort developed severe neutropenia and febrile neutropenia. This was even higher as compared to the cohort of William et al. investigating the bortezomib-BEAM (V-BEAM) regimen prior to ASCT, with 59% of the patients developing fever in neutropenia [26]. It should be considered, though, that William et al. administered lower doses of etoposide and cytarabine.

As bortezomib is known to cause peripheral neuropathy, we focused specifically on this aspect. Six patients (55%) from our bortezomib-BeEAM cohort developed peripheral neuropathy. Williams et al. documented a proportion of 33% of patients with peripheral neuropathy in their bortezomib-BEAM cohort. Differences in bortezomib dosage may provide an explanation, as bortezomib was applied at a higher dosage in some patients, whereas others received a lower dosage. Further frequent side effects in our cohort were weight loss (91%), diarrhea (100%), xerostomy (91%), and mucositis (82%). In the study of Williams et al., similar toxicities occurred but at a lower frequency than in our cohort. Considering renal toxicity, seven patients in our bortezomib-BeEAM cohort (64% of all patients) had a decline in renal function, with five of them (45% of all patients) developing acute renal failure. In contrast, William et al. described clearly lower rates of renal affection, with 9% in their bortezomib-BEAM (V-BEAM) cohort [26]. In the study of Hueso et al., similar rates of acute renal failure were described as in our cohort under HDCT with BEAM or BeEAM [8].

Considering response rates in our bortezomib-BeEAM cohort with a PFS of 64% and an OS of 64% in the last follow-up, those were even lower compared to previous studies reporting the results of Visani et al. with the BeEAM regimen, Gilli et al. also with the BeEAM regimen, and William et al. with the bortezomib-BEAM regimen [22,23,24,26]. To approximate a representative comparator arm as accurately as possible, we compared our findings with previously published studies conducted by our center investigating the BeEAM regimen. The findings of Prediletto et al., considering the toxicity of BeEAM in lymphoma patients, showed acute kidney injury in 41.1% of patients. In our findings with bortezomib addition, a decline in renal function occurred in 64% of patients. The mortality was 4.9% 100 days after ASCT in lymphoma patients receiving BeEAM combined with myeloma patients receiving bendamustine and melphalan HDCT [15]. In the present cohort, we observed a 9% mortality rate at 100-day follow-up. Mathys et al. analyzed a cohort from our center consisting of 97 MCL patients receiving BEAM (59% of patients) and BeEAM (41% of patients) as HDCT. They showed a higher CR rate of 80% at a 100-day follow-up, a higher CR rate at the last follow-up of 63%, and lower mortality at the last follow-up of 30% compared with our study [6]. In direct comparison with our cohort and patients with the BeEAM regimen treated in our center, no trend in favor of the addition of bortezomib is apparent. Thus, there was no clear outcome benefit of the new combination that may justify the increased toxicities associated with the addition of bortezomib to BeEAM. In addition to the observed toxicities that were widely consistent with the findings of the previously shown side effects of BeEAM and BEAM, we found toxicities that are typically associated with bortezomib, such as peripheral neuropathy, in a high share affecting 55% of patients. The developed neuropathies had a limiting dimension in regard to the daily lives of patients, with a median CTCAE grade of two, respectively, and two and a half in patients suffering from peripheral sensory and peripheral motor neuropathies. Nevertheless, it needs to be mentioned that the 2BeEAM regimen is an all in all feasible regimen, which may need to receive a chance for further studies considering the limited size of our cohort. Mathys et al. could show a significantly longer PFS and OS after HDCT (BEAM or BeEAM) and ASCT in MCL patients in ongoing first remission compared to relapsed MCL patients undergoing HDCT/ASCT. These results demonstrated the limitations of HDCT/ASCT, particularly for relapsing MCL [6]. Thus, there is a need for alternative therapy modalities, especially for relapsing MCL patients. Recently, CAR-T cell therapy has been approved by the FDA (2020), EMA (2020), and Swissmedic (2021) for patients with relapsed or refractory MCL after two or more lines of systemic therapy, including a BTK inhibitor [28,29,30]. In our center, we could document a high complete remission rate of 88% in the first nine patients with r/r MCL who had received CAR-T cell therapy in a real-world scenario [31]. Therefore, CAR-T cell therapy may provide an even better option for r/r MCL patients in the future.

## 5. Conclusions

Our data suggest that adding bortezomib to BeEAM HDCT in MCL does not provide a relevant improvement in outcome and goes along with increased toxicities. Therapy modalities like CAR-T-cell therapy, which has shown promising results in r/r MCL treatment, may be more promising for future evaluation.

## Figures and Tables

**Table 1:** Clinical characteristics of the patients.

General Parameters	Result
Patients, number	11
Demographic characteristics	
Males:females (ratio)	8:3 (2.7)
Median age at the time of diagnosis (years; range)	66 (46–70)
**Disease Parameters at Initial Diagnosis**	
Mantle cell lymphoma	11 (100%)
Ann Arbor stage	
I/II	0 (0%)
III	3 (27%)
IV	8 (73%)
B-symptoms at lymphoma diagnosis ^1^	7 (64%)
MIPI risk category ^1^	
Low risk	2 (18%)
Intermediate/low risk	1 (9%)
Intermediate risk	2 (18%)
High risk	4 (36%)
Morphologic grading ^2^	
Classical type	8 (73%)
Blastoid variant	2 (18%)
Average Ki-67 index ^1^ (SD)	36% (16.6)

MIPI risk category: mantle cell international prognostic index. ^1^ Unknown in two patients. ^2^ Unknown in one patient.

**Table 2:** Clinical status of the patients at the time of HDCT/ASCT.

Remission Status	Number (%)
CR	5 (45%)
PR	6 (55%)
**Prior chemotherapy**	
First line induction chemotherapy	11 (100%)
R-CHOP/R-DHAP	5 (45%)
R-CHOP/R-DHAO	6 (55%)
Second line therapy	1 (9%)
Median number of therapy lines (range)	1 (1–2)
**MIPI risk category**	
Low risk	1 (9%)
Intermediate risk	5 (45%)
High risk	5 (45%)
**Comorbidities and ECOG status**	
Clinically important comorbidities	6 (55%)
ECOG status ^1^ (range)	0 (0)
**Hematologic recovery**	
Median time (days) until ANC >0.5 G/l (range)	11 (9–13)
Median time (days) until Tc >20 G/l (range)	22.5 (13–97)

CR: complete remission; PR: partial remission; R-CHOP: rituximab, cyclophosphamide, doxorubicin, vincristine, prednisone; R-DHAP: rituximab, dexamethasone, cytarabine, cisplatin; R-DHAO: rituximab, dexamethasone, cytarabine, oxaliplatin; MIPI: mantle cell international prognostic index; ECOG: Eastern Cooperative Oncology Group. ^1^ Unknown in three patients.

**Table 3:** Hematologic and non-hematologic toxicities and infections following HDCT/ASCT.

Parameter	Number (%)
Median days in hospital (range)	27 (19–89)
Febrile neutropenia	11 (100%)
Median febrile neutropenia CTCAE grade ^1^ (range)	4 (4)
Anemia	11 (100%)
Preexisting anemia	11 (100%)
Median anemia CTCAE grade ^1^ (range)	3 (2–3)
Thrombocytopenia	11 (100%)
Median thrombocytopenia CTCAE grade ^1^ (range)	4 (4)
Preexisting thrombocytopenia	4 (36%)
New/intensified neuropathy at hospitalization	6 (55%)
New/intensified peripheral sensory neuropathy	5 (45%)
Median peripheral sensory neuropathy CTCAE grade (range)	2 (1–3)
New/intensified peripheral motoric neuropathy	2 (18%)
Median peripheral motoric neuropathy CTCAE grade (range)	2.5 (2–3)
Diarrhea	11 (100%)
Weight loss ^1^	10 (91%)
Rash	10 (91%)
Xerostomy	10 (91%)
Hypotension	9 (82%)
Nausea	9 (82%)
Mucositis	9 (82%)
Decline in renal function	7 (64%)
Fatigue	7 (64%)
Constipation	6 (55%)
Dyspnea	6 (55%)
Severe infections ^2^	3 (27%)
Infections with detected microbial pathogen	10 (91%)
Infections with detected bacterial pathogen	10 (91%)
Infections with detected fungal pathogen	6 (55%)
Infections with detected viral pathogen	2 (18%)

^1^ Unknown in one patient. ^2^ Such as sepsis, septic shock, or pneumonia.

**Table 4:** Clinical course after HDCT/ASCT.

Best Response Rate	Number (%)
100-day follow-up	
Complete remission	7 (64%)
Partial remission	3 (27%)
Death between HDCT and follow-up	1 (9%)
Last follow-up	
Complete remission	6 (55%)
Partial remission	1 (9%)
Relapse following HDCT/ASCT	1 (9%)
Death after partial remission	2 (18%)
Death after relapse after 100-day follow-up	1 (9%)
Patients with further MCL therapy after HDCT/ASCT	9 (82%)
Overall mortality	4 (36%)

## Data Availability

No data supporting the reported results are deposited elsewhere.
